# Nanobiotic formulations as promising advances for combating MRSA resistance: susceptibilities and post-antibiotic effects of clindamycin, doxycycline, and linezolid

**DOI:** 10.1039/d1ra08639a

**Published:** 2021-12-13

**Authors:** Mennatallah A. Mohamed, Maha Nasr, Walid F. Elkhatib, Wafaa N. Eltayeb, Aliaa A. Elshamy, Gharieb S. El-Sayyad

**Affiliations:** Microbiology Department, Faculty of Pharmacy, Misr International University Cairo 19648 Egypt; Pharmaceutics and Industrial Pharmacy Department, Faculty of Pharmacy, Ain Shams University, African Union Organization St. Abbassia Cairo 11566 Egypt; Microbiology and Immunology Department, Faculty of Pharmacy, Ain Shams University, African Union Organization St. Abbassia Cairo 11566 Egypt walid-elkhatib@pharma.asu.edu.eg walid-elkhatib@gu.edu.eg walid2005faisal@yahoo.com +20-2-24051107 +20-2-24051120; Department of Microbiology & Immunology, Faculty of Pharmacy, Galala University New Galala City Suez Egypt; Microbiology and Public Health Department, Faculty of Pharmacy and Drug Technology, Heliopolis University for Sustainable Development Cairo Belbes Road Cairo 11788 Egypt; Drug Radiation Research Department, National Center for Radiation Research and Technology (NCRRT), Egyptian Atomic Energy Authority (EAEA) Cairo Egypt Gharieb.S.Elsayyad@eaea.org.eg Gharieb.Elsayyad@gu.edu.eg +20-2-22749298 +20-2-22727413

## Abstract

Antimicrobial activity and post-antibiotic effects (PAEs) are both important parameters in determination of the dosage regimen of antimicrobial agents. In the present study, antimicrobial activity and PAEs of clindamycin, doxycycline, linezolid, and their nanobiotic formulations were evaluated against two methicillin resistant *Staphylococcus aureus* clinical isolates (MRSA) encoded (MRSA-S1 and MRSA-S2). Nanobiotic formulations increased the susceptibility of MRSA isolates by 4–64 folds as compared to their conventional ones. The PAE values were determined after exposure of MRSA isolates for 1 h to 10× the MICs of the tested antibiotics. The duration of PAEs were recorded after bacterial growth in Mueller Hinton broth (MHB) free from antibiotic has been restored. The PAE values for MRSA-S1 were 2.5 h for the conventional antibiotics. However, the PAEs for nanobiotics were 4 h for both clindamycin and linezolid, while 3 h for doxycycline. For MRSA-S2, linezolid and linezolid nanobiotics PAEs were 3 h. PAEs of clindamycin and clindamycin nanobiotics were 3.75 h and 4 h, respectively. Doxycycline and doxycycline nanobiotics revealed the same PAEs patterns of 3.5 h. The findings of the current study may positively influence the pharmacodynamics of the antibiotics and consequently the dosage regimen of nanobiotics as well as on their clinical outcome.

## Abstract 2

Novel nanobiotic formulations of clindamycin, doxycycline, and linezolid were evaluated for the post-antibiotic effects against biofilm forming methicillin resistant *Staphylococcus aureus* (MRSA).

## Graphical abstract

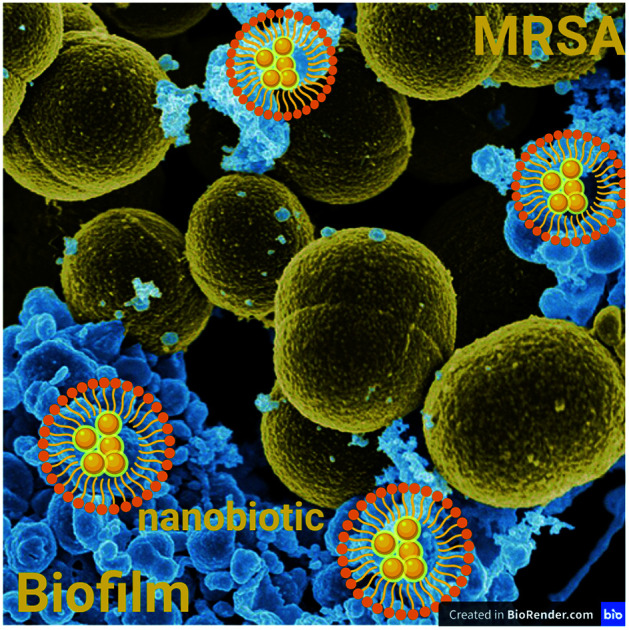

## 1. Introduction

Sir Ogston mentioned Staphylococci and their part in formation of abscess and sepsis in a variety of clinical findings and experimental studies published in 1880 and 1882.^1^ After more than 100 years, *Staphylococcus aureus* (*S. aureus*) continues to be a major dynamic and harmful pathogen for humans. Both community and hospital acquired staphylococcal infections have exponentially increased.^2^*S. aureus* is a prevalent pathogen related to severe skin and soft tissue infections, as well as pneumonia and bacteremia.^3–5^ Due to the rising incidence of antibiotic resistance and the emergence of multidrug-resistant strains, effective treatments provided for staphylococcal infections are becoming increasingly limited.^6^ Infections caused by resistant pathogens give rise to high morbidity and mortality rates, which leads to global rise in healthcare costs.^7^ This problem is even more complex when it comes to biofilm-associated infections.^8^ Bacteria in biofilm express different phenotypic characters from those expressed by their planktonic counterparts.^9,10^ Biofilms render the bacteria more resistant to the host defense mechanisms as well as to the action of antibiotics *via* many several mechanisms.^11^ Drug-resistant bacterial infections contributes to increased doses of drugs, combination of medications which elevates toxicity, long hospitalization, and higher mortality.^12^ Hence, different treatment strategies have become an essential requirement.^6,13^ However, there is no guarantee that the production of new antimicrobial drugs will timely keep up with the rapid and regular emergence of resistance by the microbial pathogens. However, nanoparticles (NPs) is one of the most promising approaches to tackle microbial resistance.^14^

Antimicrobial NPs provide many remarkable characteristics as compared to traditional antibiotics in minimizing toxic effects, overcoming resistance and lowering costs.^15^ There are also numerous nanosized drug carriers offered for the efficient administration of antibiotics by enhancing their pharmacokinetic parameters and minimizing the side effects.^16,17^

In assumption, NPs are maintained in the body for extended period's thanlow molecular weight antibiotics, which may be useful for consistent therapeutic effects.^18^

Nanoemulsions are mostly oil-in-water (o/w) or water-in-oil (w/o) where stabilization of two dispersed immiscible liquids is carried out using a suitable surfactant.^19^ The average diameter of droplets obtained is usually less than 500 nm.^20^ Coarse emulsions are associated with milky white color while small droplet size emulsions show a transparent or hazy look.^21^

Post-antibiotic effect is a pharmacokinetic factor which lead to the slow regeneration of living bacteria even after removal of the antibiotics from the growth medium.^22–25^ The period of PAE is primarily influenced by the characteristics and the concentration of the antibiotic used as well as the bacterial species. Furthermore, PAE is affected by other factors such as growth media, temperature, pH, oxygen, and the body fluid. The clinical importance of the PAE is mainly related to the implementation of antibiotic dosing strategies in medical practice. The extended PAE could allow dose reductions without decreased effectiveness, and possibly a reduction incidence of adverse effects.^23^

Drugs without PAEs, for example, that require administration more frequently than those that slow PAEs.^26,27^

Tetracyclines are wide spectrum bacteriostatic agents which inhibit protein synthesis through binding to the 30S ribosomal subunit.^28,29^ A second generation of tetracycline antibiotics is the long-acting doxycycline.^30^ It is the most commonly used tetracycline with improved lipophilic characteristics, in contrast with earlier tetracyclines.^30–33^ Clindamycin is a derivative of lincomycin, which explicitly acts on the 50S subunit of the bacterial ribosome, probably by controlling peptide chain initiation, thus inhibiting protein synthesis in bacteria.^34^ For several years, clindamycin has been recommended for treatment of serious infections caused by *S. aureus*.^35^

Linezolid is a bacteriostatic agent and protein synthesis inhibitor demonstrating excellent activity against *Staphylococcus* biofilms.^36–38^ Although linezolid has a bacteriostatic effect *in vitro*, some authors have observed that it may act as a bactericidal antibiotic *in vivo*, by inhibiting the production of staphylococcal and streptococcal toxins.^39^ Linezolid was the first commercially available oxazolidinone licensed by Food and Drug Administration in 2000 at the United States.^40^ Oxazolidinones bind to 23S ribosomal RNA of the 50S ribosomal subunit, where they prevent the formation of 70S ribosomal unit and the initiation phase of translation, inhibiting protein synthesis of bacteria.^39^ In the present study, novel nanobiotic formulations of clindamycin, doxycycline, and linezolid were evaluated for the PAEs compared with their corresponding classical antibiotics against two selected biofilm forming MRSA isolates.

## 2. Materials and methods

### 2.1 Materials

The antimicrobial agents tested in the present analysis were clindamycin, doxycycline, and linezolid. The Egyptian Group for Pharmaceutical Industries (El Obour, Cairo, Egypt) supplied Clindamycin and linezolid were provided by EIPICO (10^th^ of Ramadan City, Cairo, Egypt) provided doxycycline. From dry antibiotic powders, stock solutions were prepared at a concentration of 2560 μg mL^−1^ and stored at −20 °C according to the manufacturer's instructions. Tween 20, oleic acid, and ethanol used in nanoemulsion synthesis were purchased from Sigma Aldrich (Darmstadt, Germany).

### 2.2 Bacterial isolates

The clinical isolates used in this research are biofilm forming (MRSA-S1 and MRSA-S2) and they have been obtained from blood specimens, from patients at Ain Shams University Specialized Hospital and El-Demerdash Hospital (Cairo, Egypt), respectively. No patients have been contacted by the investigators in this study and all the patient identifiers were carefully removed by the Microbiology labs of the above mentioned hospitals before obtaining the isolates. Accordingly, the need for consent was not required by the ethics committees. The two isolates were resistant to the three standard antimicrobial agents; clindamycin, doxycycline, and linezolid. The reference strain selected for this research was *S. aureus* ATCC 25923 and was kindly and formerly provided by the United States Naval Medical Research Unit (US-NAMRU), Cairo, Egypt.

### 2.3 *In vitro* quantitative assessment of MRSA-S1 and MRSA-S2 biofilms

The two selected MRSA-S1 and MRSA-S2 were tested for formation of biofilm using 96-well flat-bottomed microtiter plates (Corning, New York, USA).^39^ Overnight *S. aureus* cultures in tryptic soy broth (TSB; Conda lab, Madrid, Spain) were diluted to the 0.5 McFarland turbidity standard in TSB (equivalent to 1.5 × 10^8^ CFU mL^−1^). Next, the bacterial suspensions were diluted to 1 : 100 in TSB to which 2% w/v glucose and 2% w/v sodium chloride were added (EL-Nasr Pharmaceuticals Chemicals Co., Cairo, Egypt). A volume of 200 microliters of these suspensions were offloaded to each of three parallel wells of the microtiter plate. Negative control wells contained sterile TSB only and *S. aureus* ATCC 25923 was used as the positive control. Plates were then incubated at 37 °C for 24 h under static conditions, the absorbance was recorded at wavelength 562 nm using ASYS expert plus microplate reader (Biochrom, England, UK) as a measure of overall growth. In turn, the culture was aspirated and plates were rinsed 3 successive times with 200 μL of 0.1% tryptone water (Sigma-Aldrich, Darmstadt, Germany) to remove non-adherent cells and then were air dried at room temperature. The residual adhesive biofilms were fixed using 200 microlitres of 95% ethanol (Sigma-Aldrich, Darmstadt, Germany) for each well and after 15 min; the plates were drained and left to air dry. The biofilms formed were stained for 5 min with 100 μL per well of membrane filtered crystal violet solution (0.1% w/v, Sigma-Aldrich, Darmstadt, Germany) at room temperature. Excess solution of crystal violet was drained and the biofilms were treated two times with 200 mL phosphate buffered saline (Sigma-Aldrich, Darmstadt, Germany). The crystal violet dye bound to the biofilm was re-solubilized with a mixture of 80% ethanol and 20% acetone (100 μL per well) and the plate was then incubated at room temperature for 20 min. The re-solubilized crystal violet was diluted with ethanol/acetone mixture (1 : 10) in a new plate and the optical density was determined. Biofilm formation was categorized as strong (OD_562_ ≥ 1.11), weak (0.22 ≤ OD_562_ < 1.11), and negative (OD_562_ < 0.22) as previously reported.^41^

### 2.4 Scanning electron microscopy (SEM) for the biofilms of MRSA isolates

Biofilms of the two MRSA isolates were examined and confirmed by SEM.^42^ Microscopy was performed at the Scanning Electron Microscopy Department, the Regional Center for Mycology and Biotechnology, Al-Azhar University, Cairo, Egypt. The images were captured using SEM JSM-5500LV (JEOL, Tokyo, Japan). Samples were examined using the secondary electron emission mode with accelerating voltages of 15 kV. The magnifications used in the examination were 5000×, 7000×, 100 00×, and 130 00×.^43^

#### 2.4.1 Preparation of MRSA biofilms for SEM examination

Biofilms were allowed to form on nutrient agar plastic Petri dishes for 48 h at 37 °C. Pieces of agar around 5 mm in diameter and 2–3 mm thick with colonies of interest were cut with a dissecting tool.^44^ To each sample, 4% glutaraldehyde (EL-Nasr Pharmaceuticals Chemicals Co., Cairo, Egypt) was used as a primary fixative in 0.1 M phosphate buffer and was added in a sufficient amount to wet the agar block. The plate was covered and left to stand for up to 24 hours at room temperature. The fixative was then decanted and replaced with an equivalent amount of rinsing buffer (0.1 M phosphate buffer) and left to stand for 30 min at room temperature.^45^ A second wash was done for removal of the primary fixative. Biofilms were post-fixed in Millonig's phosphate buffered 1% w/v osmium tetroxide (Sigma-Aldrich, Missouri, USA) for 1 h. Biofilms were then washed using distilled water and dehydrated with a sequence of ethanol solutions (25, 50, 75, then 100% each for 15 min) and air dried for 20 min. The bottoms of the medium were cut for subsequent sputter coating with gold and imaging.^46^

#### 2.4.2 Sputter coating

The aluminum stub of the microscope was placed in the storage container of the SEM specimen using the stub tweezers. Double-sided conductive carbon tape was fixed to the stub. The fixed and dried specimens were placed on the previously prepared stub. The gold to be sputtered was set as the cathode and the mounted bottoms were located on the anode to be coated. The sputter coater was operated under vacuum with argon admitted to the chamber by a fine control valve so that samples got sputter-coated with gold.^46^

#### 2.4.3 Imaging

The gold coated bottoms were placed on the holders inside the microscope specimen chamber and an electron beam was directed towards them under vacuum.^47^ The signals resulting from the different points on the sample bombarded by electrons are collected by a detector, amplified, and displayed as an image on a computer monitor.^48^

### 2.5 Preparation and characterization of nanobiotic formulations

The nanobiotics were prepared as previously mentioned.^49–52^ In short, clindamycin, doxycycline, and linezolid were solubilized in tween 20, oleic acid and ethanol mixture (Sigma-Aldrich, Darmstadt, Germany) using magnetic stirrer as stated in our previously published paper.^53^ The mixture was diluted with water dropwise till the formation of an oil in water nanoemulsion. The antibiotic concentration in the nano-formulation was 128 μg mL^−1^. All nanobiotic formulations were characterized for their size, charge, and homogeneity (Zetasizer ZS3600, Malvern Co., UK) as we described formerly.^53^

### 2.6 Determination of minimum inhibitory concentrations (MICs)

Minimum inhibitory concentrations were measured by broth microdilution method, referring to the Clinical and Laboratory Standards Institute (CLSI, 2017) for *S. aureus*.^54^ Minimum inhibitory concentrations were determined in MHB (Oxoid, England, UK) by using a two-fold serial dilution of antibiotics and nanobiotic with a 1–3 × 10^5^ CFU mL^−1^ inoculum of the tested MRSA-S1 and MRSA-S2 isolates. After 24 hours incubation at 37 °C, the MIC values were estimated.^55^ The MIC is the lowest concentration of the antibiotic which prevents visible growth of the bacteria.^56^ Determination of susceptibility were made in triplicates, and the mean of those values were reported as MIC.

### 2.7 Post-antibiotic effect determination by viable count technique

The viable count technique was used to detect the PAE as formerly mentioned.^57^ Clindamycin, doxycycline, and linezolid antibiotics as well as their nanobiotics were tested against MRSA isolates (MRSA-S1 and MRSA-S2). An overnight culture of *S. aureus* was diluted to 10^6^ CFU mL^−1^ in MHB and then incubated at 37 °C until exponential growth phase was reached.

In a shaker water bath, the cultures (10^6^ CFU mL^−1^) were incubated at 37 °C together with the antibiotics at 10× MIC for 1 hour. Simultaneously, a suspension of each organism that was not subjected to antibiotics was used as a control and was exposed to the previous procedures. After the exposure time has ended, the supernatant was centrifuged at 1200*g* for 10 min, decanted and the pellet was re-suspended in new MHB. The bacterial counts, after ten-fold serial dilution, were performed at time zero for all cultures, before and after washing and every hour up to 6 h.^58^ The duration of PAEs were obtained following the recovery of bacterial growth in antibiotic-free MHB measured as colony forming units (CFU mL^−1^) on Mueller Hinton agar (Oxoid, England, UK). The relation between time and the counts of CFU mL^−1^ were plotted graphically on logarithmic scale.^58^ The PAE was determined by the following equation: PAE = *T* − *C*, where *T* is the time needed for the counts of CFU mL^−1^ in the test culture to rise 10 folds above the count detected instantly after removal of the antibiotic and *C* is the time needed for the count of CFU mL^−1^ in the control culture to increase by 10 folds above the count obtained directly after the same procedure used on the test culture following antibiotic removal has been completed.^58^

### 2.8 Statistical analysis

The statistical examination of the obtained outcomes had been conducted applying the ONE WAY ANOVA (at *P* < 0.05) and agreed to be Duncan's multiple series studies and the least significant difference summary (LSD).^59^ The outcomes and data were reviewed and examined through SPSS software version 15.

## 3. Results

### 3.1 *In vitro* quantitative assessment of MRSA-S1 and MRSA-S2 biofilms

The biofilm activity of the two studied MRSA-S1 and MRSA-S2 showed optical densities of 1.806 and 1.893 indicating strong biofilm forming ability.^41^

### 3.2 Scanning electron microscopy of MRSA biofilms

**Fig. 1:**
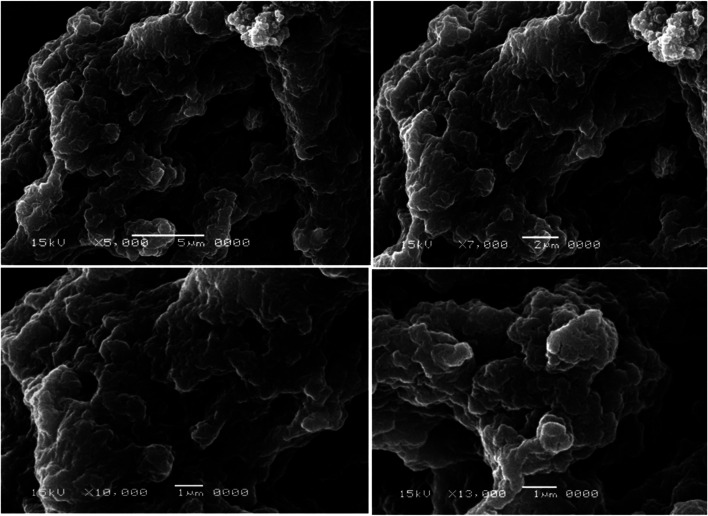
Scanning electron micrographs of biofilms of MRSA-S1 isolate. Biofilm was examined with a scanning electron microscope JSM-5500LV (JEOL, Tokyo, Japan) using the secondary electron emission mode with accelerating voltages of 15 kV. The magnifications used were 5000×, 7000×, 10 000×, and 13 000×.

**Fig. 2:**
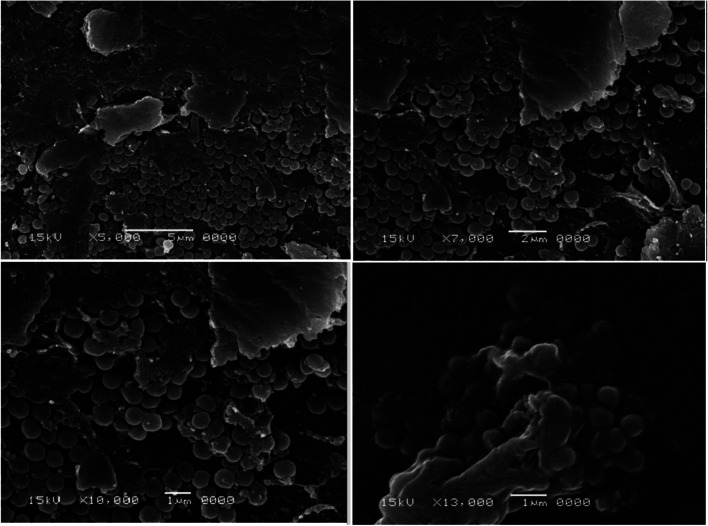
Scanning electron micrographs of biofilms of MRSA-S2 isolate. Biofilm was examined with a scanning electron microscope JSM-5500LV (JEOL, Tokyo, Japan) using the secondary electron emission mode with accelerating voltages of 15 kV. The magnifications used were 5000×, 7000×, 10 000×, and 13 000×.

The two selected MRSA isolates were visually confirmed to have biofilm by SEM as shown in figures (Fig. 1 and 2). Scanning electron micrograph of MRSA-S1 shows a developed staphylococcal biofilm and attached coccoid staphylococcal cells were evident as shown in Fig. 1. In scanning electron micrograph of MRSA-S2 isolate, the biofilm is made of clustered cocci and it was possible to partly observe the fibriform extracellular matrix. The staphylococcal cells were mostly isolated from the biofilm in certain regions of the biofilm surface (Fig. 2).

### 3.3 Characterization of the prepared nanobiotics

All nanobiotic formulations displayed a small particle size (11–14 nm), a negative charge (−10 to −13 mV), and moderate polydispersity of the formulation particles (data not shown).

### 3.4 Minimum inhibitory concentrations of the tested antibiotics against MRSA clinical isolates

According to CLSI (2017), the MIC values for all tested antibiotics against MRSA-S1 and MRSA-S2 indicated their resistance or insensitivity (Table 1). On the other hand, the MIC values for clindamycin, doxycycline, and linezolid nanobiotics showed reduction by 8, 4, and 32 folds as compared to their conventional antibiotics, respectively against MRSA-S1.

**Table 1:** Minimum inhibitory concentrations of conventional antibiotics and their nanobiotics against methicillin resistant *Staphylococcus aureus* (MRSA-S1 and MRSA-S2) clinical isolates^a^

Isolate code	MIC (μg mL^−1^)					
	Clindamycin	Clindamycin nanobiotic	Doxycycline	Doxycycline nanobiotic	Linezolid	Linezolid nanobiotic
MRSA-S1	64 (R)	8 (R)	8 (I)	2 (S)	64 (R)	2 (S)
MRSA-S2	64 (R)	1 (I)	64 (R)	2 (S)	64 (R)	4 (S)

a The cutoff values as proposed by the CLSI, (2017), MIC (μg mL^−1^) for linezolid *S* ≤ 4, *R* ≥ 8, for doxycycline *S* ≤ 4, *I* = 8, *R* ≥ 16, for clindamycin *S* ≤ 0.5, *I* from 1–2, *R* ≥ 4.

Furthermore, the MIC values for clindamycin, doxycycline, and linezolid nanobiotics revealed reduction by 64, 32, and 16 folds as compared to their conventional antibiotics, respectively against MRSA-S2. Based on these results, nanoemulsion formulations of doxycycline and linezolid rendered both of MRSA-S1 and MRSA-S2 sensitive to such antibiotics. Moreover, nanoemulsion formulation of clindamycin could increase the susceptibility of the tested isolates to this antibiotic through lowering its MIC values as compared to the conventional one (Table 1).

### 3.5 Post-antibiotic effects (PAEs) determination

**Fig. 3:**
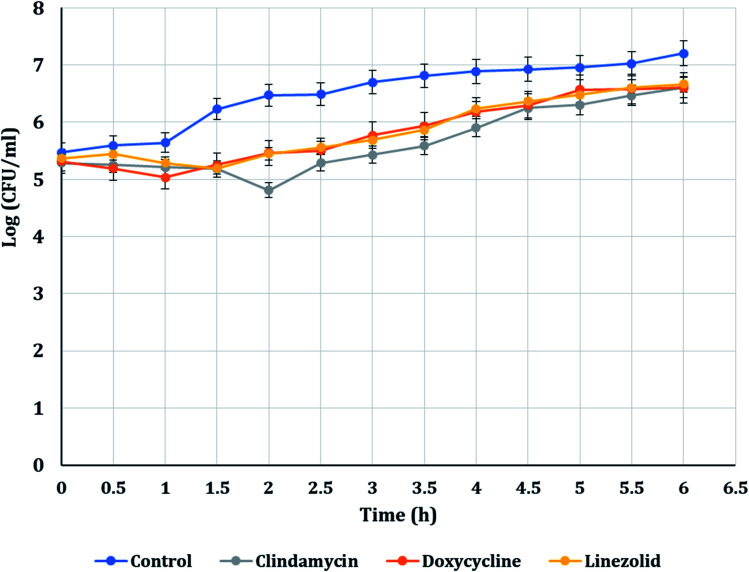
PAEs of clindamycin, doxycycline, and linezolid antibiotics on MRSA-S1.

**Fig. 4:**
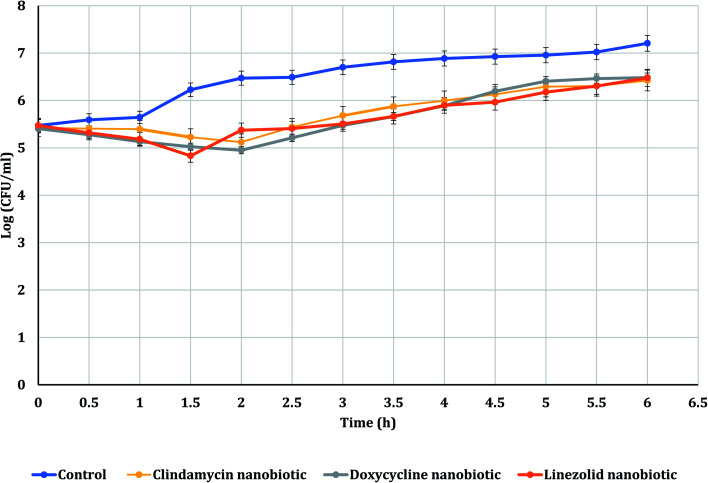
PAEs of clindamycin, doxycycline, and linezolid nanobiotics on MRSA-S1.

The PAE durations of clindamycin, doxycycline, and linezolid were determined at 10× MIC. The PAE values for MRSA-S1 obtained were 2.5 h for clindamycin, doxycycline, and linezolid while, PAEs were 4 h for nanobiotics of clindamycin and linezolid. On the other hand, the PAE was 3 h for doxycycline nanobiotic as shown in Fig. 3 and 4.

**Fig. 5:**
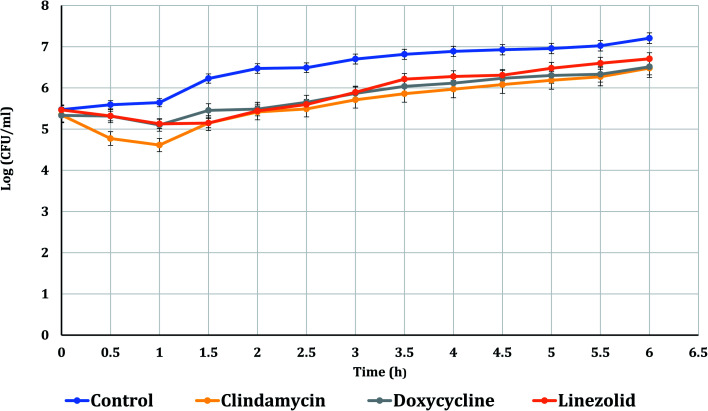
PAEs of clindamycin, doxycycline, and linezolid antibiotics on MRSA-S2.

**Fig. 6:**
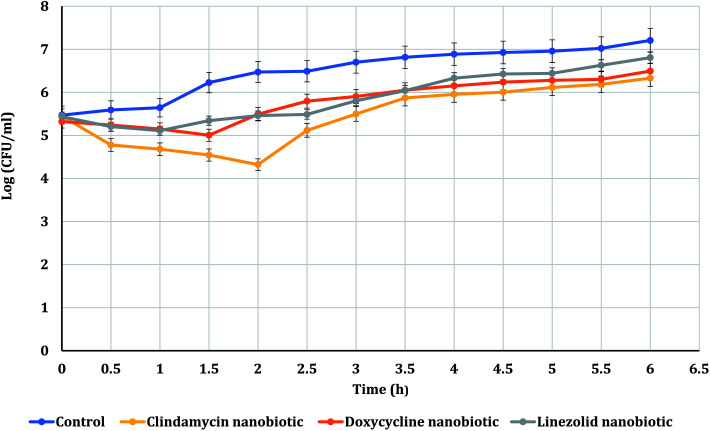
PAEs of clindamycin, doxycycline, and linezolid nanobiotics on MRSA-S2.

As illustrated in Fig. 5 and 6, for the tested MRSA-S2, linezolid and linezolid-nanobiotic exhibited similar PAE patterns of 3 h. Clindamycin and clindamycin-nanobiotic exhibited PAEs of 3.75 h and 4 h, respectively. Doxycycline and doxycycline-nanobiotic combinations exhibited similar PAEs patterns of 3.5 h. Accordingly, the findings of the current analysis revealed that the formulation of antibiotics as nanoemulsions could produce either prolonged or similar PAEs as compared to their conventional antibiotics.

## 4. Discussion

Due to the prevalence of both community-acquired and hospital-acquired staphylococcal infections, methicillin resistant *S. aureus* was selected as a focus in the current study.^60^ Another reason is their ability to form biofilms where bacteria in biofilms are 1000 times more resistant to antibiotics relative to their planktonic forms.^61–64^ For these reasons, *S. aureus* nowadays is reported among ESKAPE organisms.^13^ The ESKAPE organisms, include *Enterococcus faecium*, *S. aureus*, *Klebsiella pneumoniae*, *Acinetobacter baumannii*, *P. aeruginosa*, and *Enterobacter* spp.^65^ These alert organisms are typical causes of hospital infections and are also multidrug resistant.^66–68^ As multidrug resistant bacteria are in a race with human to develop sustainable antimicrobial agents, biofilm forming *Staphylococcus* Spp. provoked our eagerness to challenge. The pillar of successful biofilm therapy, which is viewed as a global problem, is efficient biofilm assays.^41^ Although different methods are used to assess biofilm formation,^69–71^ in the present study crystal violet assay was chosen to assess *S. aureus* biofilm production.

According to O'Toole (2011),^72^ a significant technique for studying the initial stages of biofilm formation is the microtiter plate assay. In addition, microtiter plate assay supports the development of a biofilm on the microtiter plate 's bottom and/or wall. Moreover, according to Xu *et al.* (2016),^73^ crystal violet can stain extracellular matrix, dead cells, and viable cells proving that crystal violet assay has a benefit in evaluating the overall biofilm production. Furthermore, according to Magana *et al.* (2018),^74^ one of the most widely used *in vitro* biofilm assessment techniques is crystal violet assay, enabling optical determination of biofilm thickness and total measurement of biomass, particularly in the early stages. However, in contrast to the previously mentioned advantages, owing to its high heterogeneity, crystal violet lacks reliability when the pigment binds un-specifically to negatively charged molecules or when it is unequally eluted by ethanol.^73,74^

Scanning Electron Microscope (SEM) continues to be unique in its power to analyze dimensional topography and allocation of visible structures despite the expanding range of specialized imaging instruments and the progress in types of microscopy.^75,76^ In the present study, biofilms of the two selected MRSA isolates were confirmed by SEM that has been efficiently used to capture biofilms due to its good resolution and magnification. Pattern and bulk of biofilm are essential characteristics that regulate the dynamics of substrate elimination by biofilms. SEM has been implemented on biofilms as it is an effective tool to show the fine details of living systems.^77^

Using the standard stages of fixation, post-fixation, dehydration, mounting, and sputter coating, all biofilm samples were prepared. To maintain the morphology of the biological sample, it was first fixed with aldehyde. Glutaraldehyde was used as a fixative in the current study because it permanently fixes the biofilm structure.^78^ Post-fixation with a fixative-containing heavy metal improved the strength of the cell structure and enhanced the contrast of the sample under the light source. In order to eliminate any reactive substance which could disrupt the performance of the electron microscope, the sample was dehydrated and left to dry.^79^ The dried sample was fixed to a conductive stub and covered with conductive material to minimize disruption of samples and improve topographical differentiation for enhanced imaging by SEM using secondary electron detection.^80^

Pharmacodynamics of antibiotics have been considered as vital parameters influencing the model of antibiotic intake.^81^ The PAE which is the slow bacterial regrowth after exposure to an antibiotic is a feature of pharmacodynamics studies.^82^ In the present study, viable count technique has been used to determine PAEs of different conventional antibiotics and their corresponding combinations with nanoemulsion. Methicillin resistant *S. aureus* was particularly chosen in the current study, because it is the primary cause of nosocomial infections in surgical wounds. Furthermore, biofilm forming MRSA can cause serious indwelling medical devices-associated infections.^83,84^

In the present study, the three antibiotics including linezolid, doxycycline, and clindamycin were encapsulated using nano-emulsion formulation.^85^ The nano-size gives the emulsion formulation a clear or blurry look in contrast to the coarse emulsion which appears milky white in color.^86^ Nanoemulsions were particularly designed in the present study as they are thermodynamically stable systems which are simple to make, and can dissolve hydrophobic medications, increasing the bioavailability of drugs or antibiotics.^84,87^ Compared to new antibiotics synthesis, production of antibiotic nano-systems could be cost-effective, and they are stable even with long-term storage. Antibiotic delivery using nanoparticles may afford multiple advantages including better solubility, controllable and consistent dissemination in specific tissues, sustained release, improved patient acceptance, reduced adverse effects, and increased cellular assimilation especially in biofilm forming isolates.^88^ Nanoemulsions used in this study are generally safe as previously described.^53^

The MIC values for all tested antibiotics against MRSA-S1 and MRSA-S2 isolates indicated their resistance. However, the MIC values for clindamycin, doxycycline, and linezolid nanobiotics showed reduction up to 32 folds as compared to their conventional antibiotics against MRSA-S1. Furthermore, the MIC values for clindamycin, doxycycline, and linezolid nanobiotics revealed reduction up to 64 folds as compared to their conventional antibiotics against MRSA-S2. In agreement with Allahverdiyev *et al.* (2011),^89^ the findings of the current study prove that combining antibiotics with NPs restores their inhibitory impact on different bacteria that have acquired resistance to them. As per Hussein-Al-Ali *et al.* (2014),^90^ NPs exert their antimicrobial effects either directly through their interactions with microbial cell targets, such as the cell envelope, or indirectly as potential transporters for antimicrobial agents, promoting their targeted transmission and enhanced diffusion into the bacterial cells. Compared to other antimicrobial agents, combination of bacterial protein synthesis inhibitors with nanoparticles showed higher antibacterial activity.^91^

In the present study, doxycycline exerted PAEs of 2.5 h and 3.5 h against MRSA-S1 and MRSA-S2, respectively. Doxycycline nanoemulsion exerted PAEs of 3 h and 3.5 h against MRSA-S1 and MRSA-S2 isolates, respectively. Earlier in a study performed by Cunha *et al.* (2000),^58^ it was confirmed that doxycycline exerted a dose-dependent PAE that varied between 2.5 h and 3.5 h. Recently, Sime and Roberts (2018),^92^ reported that tetracyclines exhibited a time-dependent bactericidal activity against various pathogens and could develop extended PAEs.

Clindamycin exerted PAEs of 2.5 h and 3.75 h against MRSA-S1 and MRSA-S2 isolates, respectively. Clindamycin nanoemulsion exerted PAE of 4 h against both MRSA-S1 and MRSA-S2 isolates. Results of the present study agreed with previous report, in which clindamycin exhibited an *in vitro* post antibiotic effect of 0.4–3.9 h against *S. aureus* isolates.^93^ Recently, Donaldson and Jason (2017),^94^ mentioned that clindamycin showed extended PAEs against some types of bacteria, which may be attributed to persistence of clindamycin at the ribosomal binding site. In addition, clindamycin has the ability to disrupt bacterial protein synthesis, causing changes in the bacterial cell wall, reducing bacterial adherence to host cells, and increasing intracellular destruction of susceptible organisms.^94^ Linezolid exhibited PAEs of 2.5 h and 3 h for MRSA-S1 and MRSA-S2 isolates, respectively. Linezolid nanoemulsion formulation exhibited PAEs of 4 h and 3 h, respectively. According to Sime and Roberts (2018),^92^ linezolid showed an antibacterial activity dependent on time and limited to small PAE.

Generally, nanoemulsions increase drug retention time in the desired area causing fewer adverse effects or toxicities where they act only on the wanted regions of the body.^95–97^ In nano-formulation, less drug quantity is needed due to the enhanced diffusion, improved bioavailability, increased retention time, and reduced drug loss.^98,99^ Although PAE is a crucial pharmacodynamic parameter of the antibiotic and could offer valuable clinical knowledge for a dose protocol, further studies are still needed to elucidate the mechanism. Many researches have documented that the growth kinetics, structure and biological activity of bacteria could be influenced by PAE.^100,101^ In addition, white blood cells could also exhibit stronger bactericidal activity *in vivo*. This can partly clarify why most antibiotics show longer PAE *in vivo* than *in vitro*.^3^

## 5. Conclusions

Formulations of the antibiotics as nanoemulsions could increase the susceptibility of MRSA isolates to these antibiotics through lowering the MIC values as compared to their conventional ones. Moreover, nanobiotic formulations revealed either prolonged or similar PAEs as compared to their conventional antibiotics. Consequently, this finding can influence the pharmacodynamic parameters of the antibiotic and may possess useful impacts on the dosage regimen of nanobiotics as well as on the clinical outcomes. In order to validate our findings and to determine the *in vivo* efficacy of the antibiotic formulations, more pharmacokinetic and pharmacodynamics studies are essential.

## Funding

This research has not received any specific grants from funding agencies in the public, commercial, or nonprofit sectors. The authors received no financial support for the research, authorship, and/or publication of this article.

## Compliance with ethics requirement

This article does not contain any studies involving human subjects or experimental animals.

## Consent to participate

No experimental investigation was performed on individuals within this research study.

## Consent for publication

All the co-authors are agreed for the research study publication.

## Availability of data and material

The authors stated and declare that all data is exist and available.

## Code availability

The authors stated and declare that all code is exist and available.

## Conflicts of interest

The authors stated and declare that no conflict or competing of interests.

## Supplementary Material
